# Regulated Deficit Irrigation Alters Anthocyanins, Tannins and Sensory Properties of Cabernet Sauvignon Grapes and Wines

**DOI:** 10.3390/molecules20057820

**Published:** 2015-04-29

**Authors:** Luis Federico Casassa, Markus Keller, James F. Harbertson

**Affiliations:** 1 School of Food Science, Washington State University, Wine Science Center, 2710 Crimson Way, Richland, WA 99354, USA; E-Mail: casassa.federico@inta.gob.ar; 2 Wine Research Center, Estación Experimental Agropecuaria Mendoza, Instituto Nacional de Tecnología Agropecuaria, Luján de Cuyo, 5507 Mendoza, Argentina; 3 Department of Horticulture, Washington State University, Irrigated Agriculture Research and Extension Center, 24106 N. Bunn Rd., Prosser, WA 99350, USA; E-Mail: mkeller@wsu.edu

**Keywords:** regulated deficit irrigation, Cabernet Sauvignon, wine, grape, berry size, tannins, anthocyanins, polymeric pigments, sensory properties

## Abstract

Four regulated deficit irrigation (RDI) regimes were applied to Cabernet Sauvignon grapes, which were analyzed for phenolics and also made into wine over three consecutive growing seasons. Relative to an industry standard regime (IS), yield was reduced over the three years by 37% in a full-deficit (FD) regime and by 18% in an early deficit (ED) regime, whereas no yield reduction occurred with a late deficit (LD) regime. Relative to IS, skin anthocyanin concentration (fresh weight basis) was 18% and 24% higher in ED and FD, respectively, whereas no effect was seen in LD. Seed tannin concentration was 3% and 8% higher in ED and FD, respectively, relative to the other two RDI regimes, whereas seed tannin content (amount per berry) was higher in IS than in FD. There were no practically relevant effects on the basic chemistry of the wines. The finished wines showed concentrations of tannins and anthocyanins that generally mirrored observed differences in skin and seed phenolic concentrations, although these were amplified in FD wines. Descriptive sensory analysis of the 2008 wines showed that FD wines were the most saturated in color, with higher purple hue, roughness, dryness and harshness, followed by ED wines, whereas IS and LD wines were less saturated in color and with higher brown and red hues. Overall, FD and ED seemed to yield fruit and wine with greater concentrations of phenolics than IS and LD, with the additional advantage of reducing water usage. However, these apparent benefits need to be balanced out with reductions in crop yields and potential long-term effects associated with pre-véraison water deficits.

## 1. Introduction

Arid climates afford grape growers unique control over the vineyard water status, and ultimately over the vines’ vegetative and reproductive growth. In arid regions with Mediterranean-type climates where annual rainfall is typically below 250 mm, irrigation can be managed through drip irrigation systems with the aim of controlling shoot vigor and reproductive growth [1,2]. The Columbia Valley of central Washington (USA) is one such area due to extremely low annual rainfall (~200 mm), which prohibits effective grape growing without irrigation [3,4]. Summers in this region are warm, with an average of 13 days with temperatures >35 °C and about 3 days with temperatures >40 °C [3]. The growing season is short, with an average frost-free period of 160 days; winters are cold whereby temperatures below −20 °C can be reached [3,4]. During the growing season, daily temperature swings can reach ~18 °C, thus allowing the berries to ripen under conditions of warm days and cool nights [3].

Regulated deficit irrigation (RDI) consists of applying short episodes of water restriction, typically starting after bloom (anthesis), whereby irrigation water is supplied at amounts below those lost to vineyard or crop evapotranspiration (ET_c_) [3]. Reported benefits of RDI include reduced vineyard water use [3,5] and control of canopy vigor, berry size and yield, which leads to changes in fruit chemistry and wine composition [3,6,7]. However, compositional changes induced in the grapes do not consistently translate into the wines, and discrepancies between grape and wine chemistries have been often reported [8,9]. This is because quality-relevant compounds are compartmentalized in cells within the berry tissues that, after cell disruption during crushing operations, can undergo enzymatic and non-enzymatic alterations, as well as covalent and non-covalent interactions with themselves and other components of the must or wine matrix.

Typical applications of RDI regimes under field conditions and commercial vineyards are not very severe, with industry standards ranging from replenishment of 60% to 70% ET_c_ [3,10]. Under those conditions, fruit reaches full maturity with limited effects on soluble solids accumulation (*i.e.*, Brix) and pH. Indeed, the effect of RDI on berry composition is primarily on skin-based compounds including free aroma components, glycosylated-aroma precursors, as well as phenolic compounds. Furthermore, sensory studies indicate a positive effect of RDI on wine aroma. For instance, descriptive analysis of Cabernet Sauvignon wines showed that RDI positively impacted black and red fruit aromas [10].

From a chemical and sensory standpoint, the two most relevant phenolic classes in grapes and wines are anthocyanins and tannins. These are synthesized via the phenyl-propanoid biosynthetic pathway [11], which is modulated by both biotic and abiotic factors, irrigation practices being among them [12]. It has been shown that water deficit effectively up-regulates the expression of genes affecting the biosynthesis of both anthocyanins and tannins [13,14]. Anthocyanins are localized in the berry skin of dark grape cultivars (and in the mesocarp of teinturier varieties) and are present as glycosylated monomers of malvidin, cyanidin, petunidin, peonidin, delphinidin, and in trace amounts of pelargonidin. Anthocyanins are responsible for the color of red wines but are tasteless or indistinctly flavored [15]. Tannins are present in seeds, skins and stem/rachis tissues as oligomers and polymers of four flavan-3-ol subunits: (+)-catechin, (−)-epicatechin, (−)-epigallocatechin, and (−)-epicatechin-3-O-gallate [16]. In red wines, tannins are responsible for the sensation of astringency, which is mediated by the precipitation of salivary proteins by the tannins [17]. However, wine tannins can also elicit bitterness [18]. Furthermore, tannins also modulate wine color via their covalent reaction with anthocyanins to form polymeric pigments, which are orange or brick-red pigments with astringent properties [19].

Changes in berry size are readily induced by irrigation practices [3,5,20,21], and these changes can in turn affect the phenolic composition of grapes [22]. Reductions in berry size are considered desirable from a winemaking perspective, because the surface to volume ratio of small berries is higher than that of larger berries. However, the question remains whether the desirable effects of RDI on grape and wine phenolics occur because of enhanced biosynthesis (*i.e.*, on a per berry basis), or due to enhanced concentration (*i.e.*, on a fresh weight basis). Another question is to what extent these changes in the grapes translate into the resulting wines. In the present experiment, four RDI protocols were applied to a commercial vineyard of cv. Cabernet Sauvignon located in the Columbia Valley American Viticultural Area (AVA). Our aim was to evaluate the influence of both timing and extent of these RDI regimes on grape and wine phenolic composition and sensory properties.

## 2. Results and Discussion

### 2.1. Weather, Irrigation and Vine Canopy

**Figure 1:**
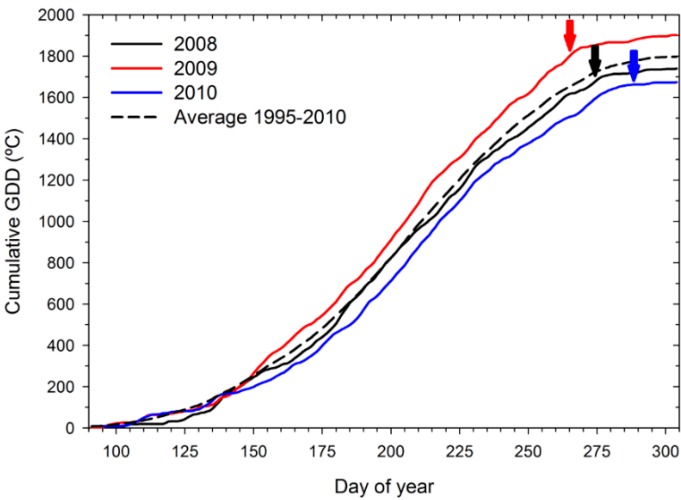
Growing degree days (GDD) accumulation (base 10 °C) from April 1st (Day 91) to October 31st (Day 304) during three growing seasons and long-term average (1995–2010) in field-grown, own-rooted Cabernet Sauvignon grapevines in the Columbia Valley, WA (USA). Black, red and blue arrows indicate the time of harvest in 2008, 2009, and 2010, respectively.

As shown in Figure 1, heat accumulation (growing degree days, GDD) during the 2008 growing season was close to the long-term average, whereas 2009 was considerably warmer and 2010 much cooler than the long-term average. There were 12 days with maximum temperatures above 35 °C in 2008 *versus* 18 days in 2009 and 7 days in 2010. Each year, only one of these hot days occurred during the fruit ripening period, the remainder occurred between fruit set and *véraison* (onset of ripening). Precipitation during the three growing seasons was minimal albeit with seasonal variations: twenty-eight millimeters (2008), 65 mm (2010) and 138 mm (2009) (Supplemental Figure S1). The RDI regimes worked as intended, although there were some differences in irrigation water supply among years (Supplemental Figure S2). Overall, water supply was similar for the industry standard (IS) and late-deficit (LD) regimes until *véraison* and diverged during ripening. The early-deficit (ED) and full-deficit (FD) regimes received similar amounts of water but less than IS and LD until *véraison*; thereafter ED and FD also differed from each other. During ripening, water supply in ED was similar to IS, and LD was similar to FD. On average over the three years, ED, LD and FD reduced the total irrigation water supply by 49%, 27% and 67% relative to IS, respectively (Supplemental Figure S2). However, total water supply in 2008 was substantially lower than in 2009 and 2010. This may be explained partly by differences between growing seasons in heat accumulation (highest in 2009) and partly by differences in canopy size (greatest in 2010; see Table 1). Indeed, both warmer conditions and larger canopies typically lead to greater evaporative demand. Despite the differences in water supply among RDI regimes, there were few differences in vine canopy characteristics; only FD reduced shoot numbers and pruning weight relative to the other RDI regimes (Table 1). While a reduction in plant vigor under the relatively severe FD regime was consistent with earlier research [3,23,24,25], the similarity in canopy characteristics among the other regimes was not surprising. Differential water supply did not start until control of shoot growth had been achieved, that is, shoots grew only insignificantly while the treatments were in place. Notably, however, the lack of RDI treatment × season interaction on leaf layer and shoot number, pruning weight and cluster exposure indicates that the RDI regimes impacted canopy development independently of the growing seasons.

**Table 1:** Two-way ANOVA of the effect of a regulated deficit irrigation (RDI) regime and growing season on canopy characteristics of field-grown, own-rooted Cabernet Sauvignon grapevines in the Columbia Valley, WA, USA.

RDI Treatment	Leaf Layer Number	Sun-Exposed Clusters (%)	Shoot Number (/m)	Pruning Weight (kg/m)	Cane Weight (g)
IS ^†^	2.5 ± 0.2 ^‡^	45 ± 7.2	20 ± 0.7 a	0.39 ± 0.02 a	20 ± 0.8 a
ED	2.6 ± 0.3	50 ± 9.2	21 ± 0.8 a	0.38 ± 0.03 a	18 ± 1.1 ab
LD	2.7 ± 0.2	60 ± 6.9	20 ± 0.8 a	0.41 ± 0.03 a	20 ± 0.9 a
FD	2.2 ± 0.1	46 ± 7.0	18 ± 0.7 b	0.31 ± 0.02 b	17 ± 0.9 b
**Season**					
2008	2.6 ± 0.1 a	51 ± 5.7	18 ± 0.4 b	0.36 ± 0.01 b	20 ± 0.5 a
2009	2.1 ± 0.2 b	54 ± 7.0	17 ± 0.7 b	0.31 ± 0.03 b	17 ± 1.1 b
2010	2.6 ± 0.2 a	46 ± 6.9	23 ± 0.7 a	0.44 ± 0.03 a	19 ± 0.9 ab
**RDI × Season interaction**	ns	ns	ns	ns	ns

^†^ IS: industry standard; ED: early deficit (fruit set to *véraison*); LD: late deficit (*véraison* to harvest); FD: full season deficit (fruit set to harvest); ^‡^ Means followed by different letters differ significantly at *p* < 0.05 by Duncan’s new multiple range test; ns: Not significant.

### 2.2. Yield Components

Relative to IS, yield was reduced, on average over the three years, by 37% in FD and by 18% in ED, whereas no yield reduction occurred with LD (Table 2). Consistent with earlier research [26], the limited yield under ED and FD was mostly due to a reduction in berry weight, which in turn reduced cluster weight. Although approximately half the volume gain in grape berries occurs after *véraison*, it has long been known that water deficit during early berry development limits berry growth, whereas *post*-*véraison* water deficit generally has little effect on berry growth [26]. Nevertheless, the 12% reduction in berry weight in ED and FD relative to IS and LD did not account fully for the 26% decrease in cluster weight in FD (Table 2). A reduction in cluster weight in the ED and FD treatments may have also arisen from a decrease in the number of berries per cluster, as a prolonged water deficit may have limited inflorescence branching, thereby decreasing the number of berries per cluster [27]. However, the number of berries per cluster was unaffected by the RDI treatments. Additionally, there was no influence of the RDI treatments on the number of clusters per vine in accordance with other studies [27,28]. Overall, these results suggest that bud fruitfulness (inflorescence primordia/bud) was not affected by the RDI regimes herein studied.

**Table 2:** Two-way ANOVA of the effect of RDI regime and growing season on yield components of field-grown, own-rooted Cabernet Sauvignon grapevines in the Columbia Valley, WA, USA.

RDI Treatment	Clusters/Vine	Cluster Weight (g/Cluster)	Berries/Cluster	Berry Weight (g)	Yield (t/ha)
IS ^†^	71 ± 3.3	82.1 ± 2.2 a ^‡^	87 ± 10 a	0.94 ± 0.03 a	8.71 ± 0.38 a
ED	69 ± 3.3	73.2 ± 3.1 b	87 ± 7 a	0.83 ± 0.03 b	7.11 ± 0.26 b
LD	72 ± 3.1	78.6 ± 2.3 a	85 ± 3 a	0.91 ± 0.02 a	8.50 ± 0.40 a
FD	63 ± 3.3	59.8 ± 2.3 c	74 ± 7 a	0.80 ± 0.03 b	5.51 ± 0.32 c
**Season**					
2008	75 ± 2.2 b	71.6 ± 1.5 b	87 ± 2 a	0.82 ± 0.02 b	8.18 ± 0.25 a
2009	82 ± 2.7 a	58.7 ± 1.8 c	70 ± 4 b	0.83 ± 0.03 b	7.47 ± 0.36 a
2010	49 ± 2.1 c	89.1 ± 2.2 a	92 ± 5 a	0.96 ± 0.02 a	6.69 ± 0.32 b
**RDI × Season interaction**	0.0411	0.0352	0.0064	0.0358	0.0481

^†^ IS: industry standard; ED: early deficit (fruit set to *véraison*); LD: late deficit (*véraison* to harvest); FD: full season deficit (fruit set to harvest); ^‡^ Means followed by different letters differ significantly at *p* < 0.05 by Duncan’s new multiple range test.

Yield components were also subject to significant annual variation. The cluster number was highest in 2009 and lowest in 2010, whereas the opposite trend was observed for cluster weight (Table 2). Berries were heaviest in 2010, suggesting that the low cluster number in 2010 triggered compensatory changes in berry number and berry weight [29]. In addition, the relatively cool temperatures in 2010 may have further promoted berry growth [3,30]. Despite this partial yield component compensation, the overall yield was lowest in 2010 (Table 2). The significant RDI treatment × season interaction on yields was the result of the absence of an irrigation treatment effect in 2008 (data not shown).

### 2.3. Fruit Composition

Fruit soluble solids were highest in IS, and titratable acidity (TA) was lowest in LD, but the RDI regimes did not alter fruit pH (Table 3). These data are consistent with previous reports on the effects of RDI [3,24,31]. Despite the decrease in soluble solids under the more severe RDI regime (*i.e.*, FD), the fruit in our study always reached Brix levels at or above those required for standard winemaking of premium fruit. Fruit composition varied much less from year to year than did yield (Table 2 and Table 3). The lower pH in 2010 was most likely a consequence of the cooler temperatures during that growing season [29].

**Table 3:** Two-way ANOVA of the effect of RDI regime and growing season on soluble solids, titratable acidity and pH of fruit at harvest of field-grown, own-rooted Cabernet Sauvignon grapevines in the Columbia Valley, WA, USA.

RDI Treatment	Soluble Solids (Brix)	Titratable Acidity (g/L)	pH
IS ^†^	27.4 ± 0.2 a ^‡^	5.60 ± 0.11 b	3.74 ± 0.03
ED	26.4 ± 0.4 b	5.34 ± 0.11 b	3.75 ± 0.02
LD	26.4 ± 0.4 b	5.99 ± 0.18 a	3.68 ± 0.03
FD	26.3 ± 0.3 b	5.36 ± 0.13 b	3.70 ± 0.02
**Season**			
2008	27.2 ± 0.2 a	5.54 ± 0.08	3.74 ± 0.02 a
2009	26.0 ± 0.4 b	5.71 ± 0.12	3.76 ± 0.02 a
2010	26.5 ± 0.2 b	5.47 ± 0.19	3.64 ± 0.02 b
**RDI × Season interaction**	0.0157	<0.0001	0.0213

^†^ IS: industry standard; ED: early deficit (fruit set to *véraison*); LD: late deficit (*véraison* to harvest); FD: full season deficit (fruit set to harvest); ^‡^ Means followed by different letters differ significantly at *p* < 0.05 by Duncan’s new multiple range test.

**Figure 2:**
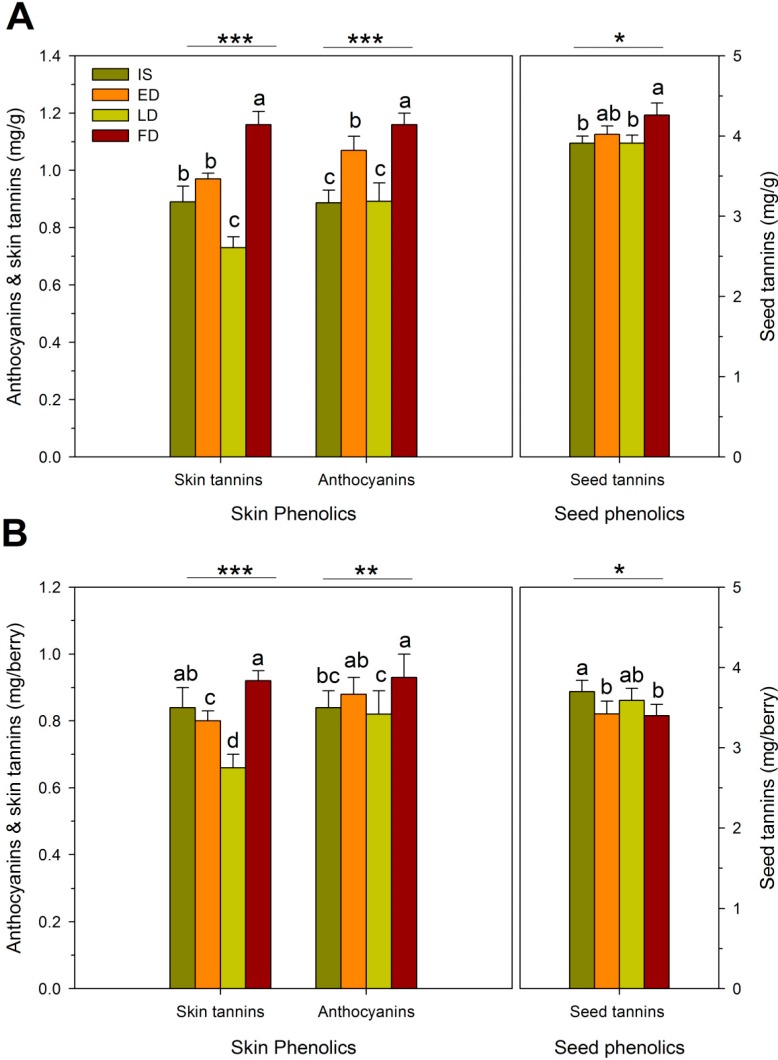
Skin and seed phenolics at harvest expressed on (**A**) fresh weight basis, and (**B**) per berry basis as affected by four RDI treatments during three growing seasons in field-grown, own-rooted Cabernet Sauvignon grapevines in the Columbia Valley, WA (USA). IS: industry standard; ED: early deficit (fruit set to *véraison*); LD: late deficit (*véraison* to harvest); FD: full season deficit (fruit set to harvest). *, **, and *** indicate significant differences for Duncan test and *p* < 0.05, *p* < 0.01 and *p* < 0.001, respectively.

The RDI regimes affected both the concentration (amount per unit fresh weight) and the absolute content (amount per berry) of skin and seed phenolics. Relative to IS, skin anthocyanin concentration was 18% and 24% higher in ED and FD, respectively, but no effect was seen in LD (Figure 2A). These changes were in line with the observed reduction in berry weight (Table 2). It has been hypothesized that pre-*véraison* RDI may increase anthocyanin concentration by selectively decreasing mesocarp rather than skin growth [22,32], or conversely, by selectively increasing the absolute mass of skin tissue [33]. An increase in concentration is also possible simply because the surface of a sphere increases with the square of the radius, while volume increases with the cube of the radius; thus, smaller berries have a relatively higher surface to volume ratio than larger ones [34]. Whichever the case, previous studies have shown that water deficit increased the concentration of skin anthocyanins in Cabernet Sauvignon [35,36] and Merlot [8,37], which concurs with the results presented herein. Skin tannin concentration increased by 23% in FD, but no effect was observed for ED, and an 18% decrease was observed in LD, relative to IS. The reduction in the concentration of skin tannins in LD occurred in all three years (data not shown). The reasons for this effect are currently unknown, especially considering that plant vigor, canopy density, cluster exposure and yield did not differ between IS and LD (Table 1 and Table 2).

Seed tannin concentration was 3% and 8% higher in ED and FD, respectively, relative to the other two RDI regimes (Figure 2A). As with anthocyanins, the concentration effect on seed tannins was partially due to the lower berry weight in FD (Table 2). In previous studies, however, water deficit did not alter the concentration of seed tannins in Shiraz [22] and Cabernet Sauvignon [35] in spite of its impact on berry weight. Seed tannin concentration is also determined by seed weight and the number of seeds per berry [38]. There are conflicting reports on how vine water status affects seed weight and number, as some studies found increases due to water deficit [39] and some have found no effect [40].

The difference in skin tannin concentration between RDI regimes was mostly preserved when data were analyzed as tannin content (amount per berry). However, for seeds, IS had a higher seed tannin content than FD (Figure 2B). This suggests that while a severe water deficit such as FD might have limited seed tannin biosynthesis [36], the simultaneous impact of the deficit on lowering berry size overrides this effect, thereby increasing overall seed tannin concentration. The RDI effect on anthocyanin content was similar to, but less pronounced than, the effect on anthocyanin concentration. This implies that, in the case of FD, increased anthocyanins were not only the result of a reduction in berry size but also of enhanced biosynthesis [41]. Enhanced expression of some key genes involved in anthocyanin biosynthesis, transport and vacuolar sequestration is at least partially mediated by the hormone abscisic acid (ABA), which serves as a drought-stress signal in plants [7,40,41,42,43].

**Figure 3:**
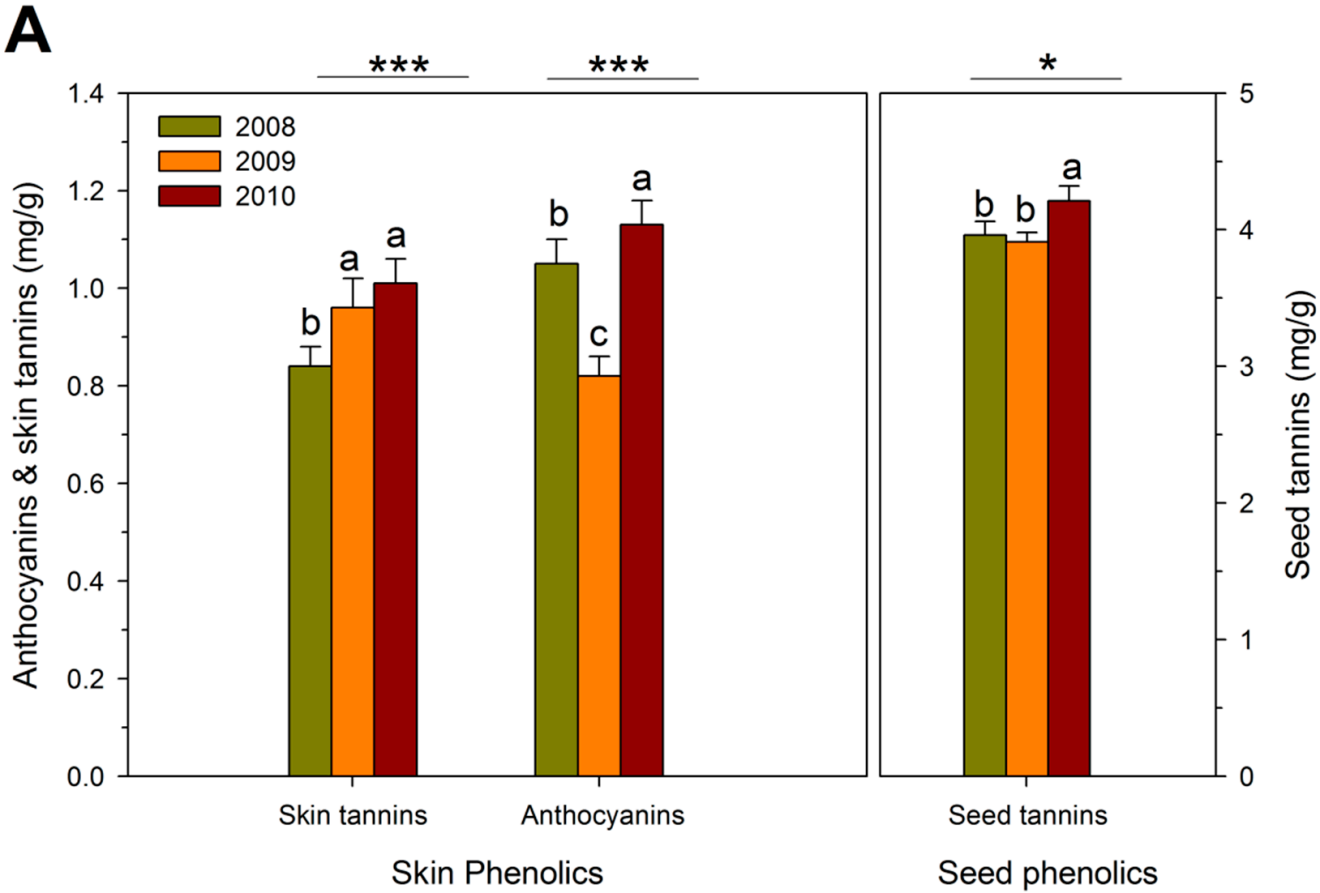

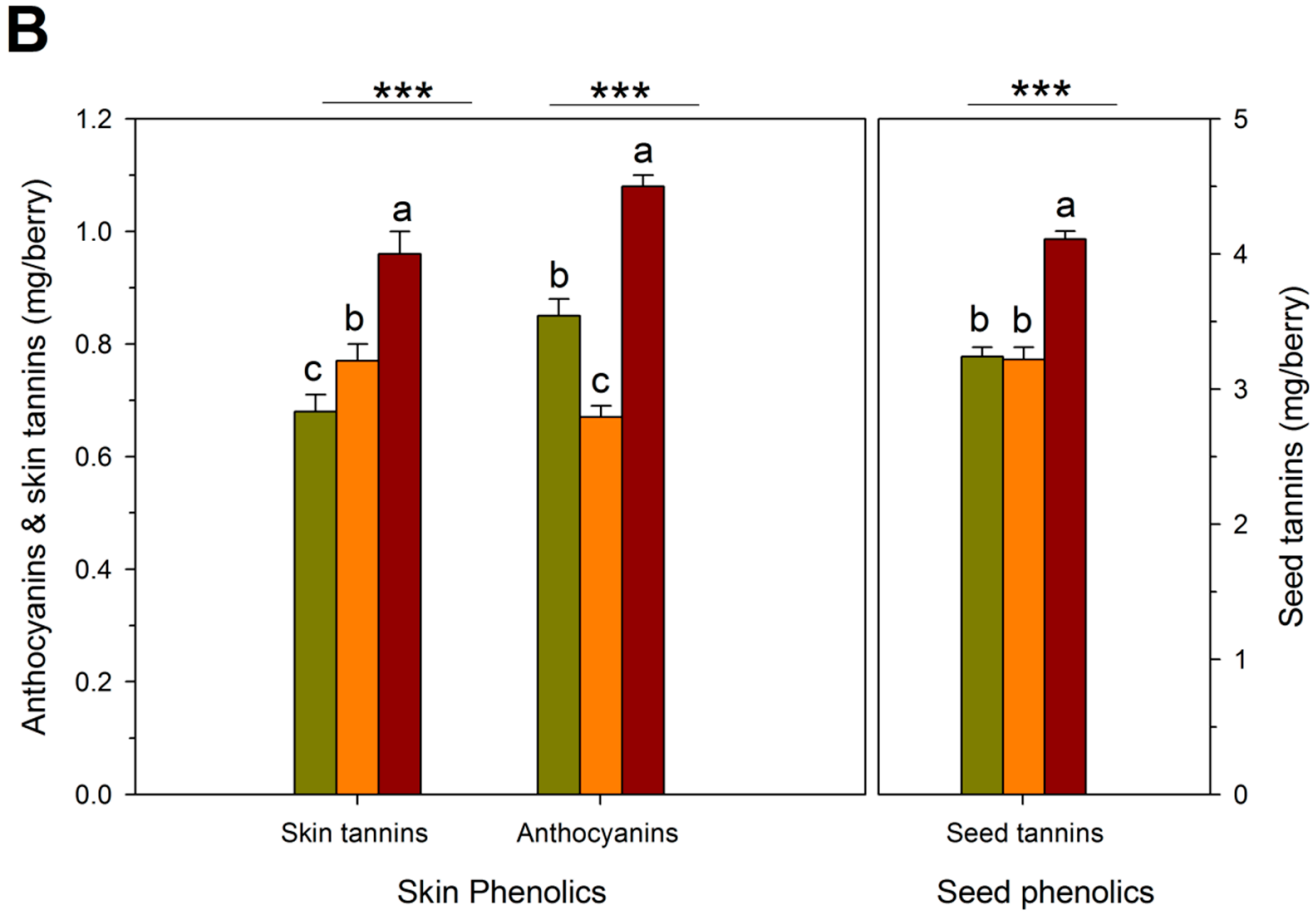
Skin and seed phenolics at harvest expressed on (**A**) fresh weight basis; and (**B**) per berry basis during three growing seasons in field-grown, own-rooted Cabernet Sauvignon grapevines in the Columbia Valley, WA (USA). *, **, and *** indicate significant differences for Duncan test and *p* < 0.05, *p* < 0.01 and *p* < 0.001, respectively.

The variations in tannin content and concentration were due approximately equally to the RDI regimes and to seasonal variations, while anthocyanin accumulation was clearly dominated by seasonal variations. The concentrations of anthocyanins and skin and seed tannins were highest after the cool 2010 growing season (Figure 3A), despite the greater berry size in 2010. The lowest anthocyanin concentration was achieved in the comparatively warmer 2009 growing season. Similarly, the anthocyanin content per berry was highest in 2010 and lowest in 2009, and seasonal variation in tannin contents paralleled the differences in concentration (Figure 3B). It might be argued that the comparatively lower yields in 2010 may be associated with enhanced biosynthesis of phenolics. However, the relationship between yield and grape phenolic composition is tenuous, and 2010 was also an unusually cool growing season. Low anthocyanins in 2009 may have been caused by reduced anthocyanin biosynthesis [44] and/or by enhanced anthocyanin catabolism due to high temperatures [45]. Although the seasonal variation in anthocyanin content may be explained by variation in growing season temperatures, the reasons for enhanced biosynthesis of skin and seed tannins during the cool 2010 season are currently unknown.

### 2.4. Wine Composition

Table 4 shows the basic chemistry of the wines with the separate effects of the RDI regimes and the growing seasons. There was no effect of the RDI regimes on the ethanol content of the wines, in spite of minor differences in fruit soluble solids (Table 3). As a standard practice followed by the winery, musts were watered-back to a target ethanol of 14.0% (*v*/*v*) upon crushing to avoid stuck or sluggish fermentations, so potential differences in ethanol content were forcibly minimized. Even though titratable acidity was adjusted to 7 g/L in the initial musts, the finished wines showed differences in both acidity and pH. Titratable acidity was significantly higher in ED and FD, and accordingly, the pH was lower in ED and FD relative to the other two RDI regimes. However, given the relatively minor effect of the RDI regimes on acidity (maximum variation 0.2 g/L) and pH (maximum variation 0.1 pH units), these differences are unlikely to be of sensory relevance.

The effects of the growing seasons on the basic chemistry of the wines were of higher magnitude than those caused by the RDI regimes. For example, the pH of the wines reflected fruit pH at harvest (Table 3). The 2010 growing season had the lowest ethanol content and pH relative to 2008 and 2009. There were no interactive effects between the RDI regimes and growing seasons.

**Figure 4:**
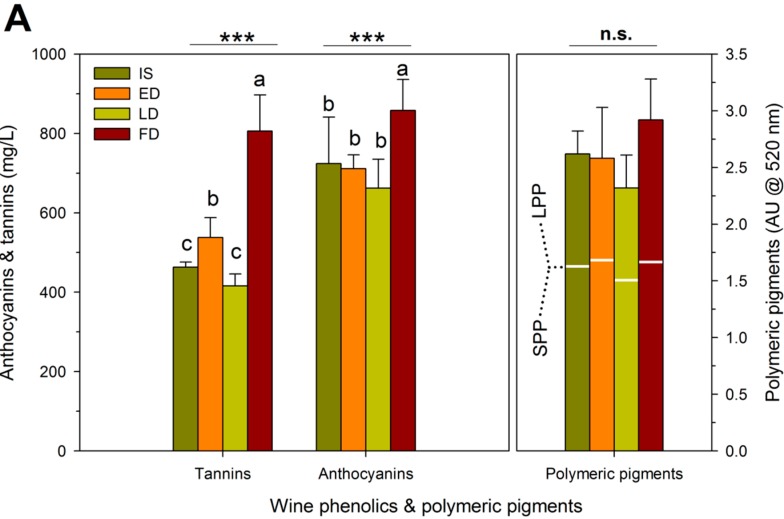

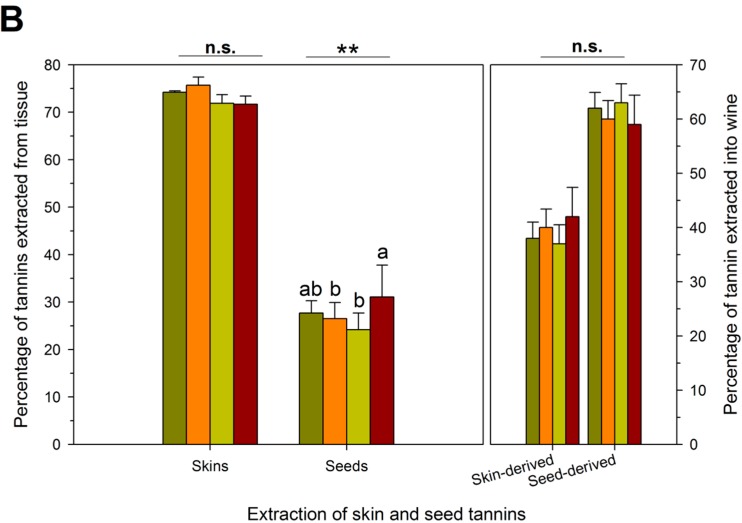
(**A**) Wine phenolics and polymeric pigments; and (**B**) extraction of skin and seed tannins as affected by four RDI treatments during three growing seasons in field-grown, own-rooted Cabernet Sauvignon grapevines in the Columbia Valley, WA (USA). IS: industry standard; ED: early deficit (fruit set to *véraison*); LD: late deficit (*véraison* to harvest); FD: full season deficit (fruit set to harvest). *, **, and *** indicate significant differences for Duncan test and *p* < 0.05, *p* < 0.01 and *p* < 0.001, respectively. N.s: not significant.

The amount of skin and seed tannin extracted into wine was calculated based on skin and seed tannins recovered in the pomace after fermentation and the concentration of skin and seed tannins measured in the fruit. Additionally, the proportion of skin- or seed-derived tannins extracted into wine was calculated as the difference between what was found in either the skins or the seeds at harvest and the amount left in the pomace and then dividing by the estimated amount of tannin extracted [46]. The finished wines showed concentrations of tannins and anthocyanins that generally mirrored observed differences in skin and seed phenolic concentrations, although the concentrations were greater in FD wines. For example, wine tannins were 42% higher in FD relative to IS. Likewise, tannins were 14% and 23% higher in ED relative to IS and LD, respectively (Figure 4A). Anthocyanins were higher in FD wines, and there were no differences in wine anthocyanin concentration among the other three RDI regimes. A previous report from our group which sourced Cabernet Sauvignon grapes from the same experimental plot found that the extraction patterns of both anthocyanins and tannins during the maceration process were unaffected by four RDI regimes [6], but quantitative differences were observed. In that study, the 25% ET_c_ wines retained more anthocyanins at day 400 after crush. In the present study, wine anthocyanins and tannins were determined at a single point in the finished wines, but similar to [6], anthocyanins as well as tannins were higher in the FD wines.

**Table 4:** Two-way ANOVA of the effect of RDI regime and growing season on pH, titratable acidity and ethanol of finished wines made from fruit of field-grown, own-rooted Cabernet Sauvignon grapevines in the Columbia Valley, WA, USA.

RDI Treatment	Ethanol % (*v*/*v*)	Titratable Acidity (g/L)	pH
IS ^†^	14.0 ± 0.2	5.51 ± 0.21 b	3.82 ± 0.08 a ^‡^
ED	13.9 ± 0.1	5.67 ± 0.19 a	3.74 ± 0.07 b
LD	14.1 ± 0.1	5.51 ± 0.21 b	3.76 ± 0.07 ab
FD	13.9 ± 0.1	5.71 ± 0.18 a	3.72 ± 0.06 b
**Season**			
2008	14.2 ± 0.1 a	5.38 ± 0.04	3.78 ± 0.02 b
2009	14.0 ± 0.1 a	5.22 ± 0.09	3.94 ± 0.21 a
2010	13.7 ± 0.1 b	5.19 ± 0.03	3.56 ± 0.01 c
**RDI × Season interaction**	ns	ns	ns

^†^ IS: industry standard; ED: early deficit (fruit set to *véraison*); LD: late deficit (*véraison* to harvest); FD: full season deficit (fruit set to harvest); ^‡^ Means followed by different letters differ significantly at *p* < 0.05 by Duncan’s new multiple range test; ns = Not significant.

Figure 4A also shows the effect of the RDI regimes on the polymeric pigment content of the wines. The total polymeric pigment content represents a summation of small (SPP) and large polymeric pigments (LPP), which are also indicated in Figure 4A. SPP are formed by reaction of anthocyanins with miscellaneous compounds, including acetaldehyde, pyruvic acid, and flavan-3-ol monomers or dimers [47], resulting in low molecular size pigments that do not precipitate the BSA protein used in the method to assess grape and wine tannins. Conversely, LPP are pigmented tannins that precipitate with BSA, and they reportedly contribute to perceived astringency [48,49]. The RDI regimes had no effect on the total polymeric pigment content of the wines. However, the LPP content was significantly higher in FD wines. Previous studies had shown that higher tannin levels during maceration led to significantly greater formation of LPP [46,48], which is consistent with the higher tannin levels in the FD wines. Previously, the evolution of SPP and LPP during maceration and aging of Cabernet Sauvignon wines of four RDI regimes (100% ET_c_, 70% ET_c_, 25/100% ET_c_ and 25% ET_c_), also showed a higher LPP content in the 25% ET_c_ wines at press and after 400 days post-crushing [6]. Moreover, the LD wines with the lowest tannin content (together with IS wines) also had the lowest LPP content. These results confirm that there is a positive relationship between wine tannin content and the formation of LPP during winemaking.

To obtain additional information about wine tannin concentrations, the proportions of skin- and seed-derived tannins extracted into wine were estimated (Figure 4B). Tannin extraction from skins ranged from 72% to 76%; however, there were no differences in skin tannin extraction among the four RDI regimes. Tannin extraction from seeds ranged from 24% to 31%, with FD showing higher extraction than ED and LD. These results suggest that skin tannin extraction is far more complete than seed tannin extraction, as previously shown elsewhere [46,49]. Reported percentages of extracted tannins ranged from 33% to 68% in skins and from 4% to 56% in seeds [46,50].

The skin-derived tannins accounted for 37% to 42% of the wine tannin content whereas the seed-derived tannins accounted for 59% to 62% with no effect of the RDI regimes on these percentages. These results are different from the above reported tannin extraction from skins and seeds. This apparent discrepancy can be explained by the fact that grape berries contain much more seed tannin than skin tannin (approximate ratio 4:1, see Figure 2). Previous reports indicated that seed tannins represented 52% to 80% of the wine tannin mass [10,46,49,51]. In a previous study from our group, the proportions of skin- and seed-derived tannins retained in Cabernet Sauvignon wine were found to be unaffected by the RDI regimes (100% ET_c_, 70% ET_c_, 25/100% ET_c_ and 25% ET_c_) [6]. However, the winemaking practice known as extended maceration can favor tannin extraction from seeds over skins [6,46,49,51]. All wines in the present experiment were produced with a standard maceration period of 7 days, and thus a shift in the proportions of skin- and seed-derived tannins was not anticipated. This was generally the case, with the sole exception of a greater amount of tannins extracted from seeds in FD wines. The higher extraction of seed tannins in FD in turn explains the higher total tannin concentration in FD wines. Furthermore, this differential effect of seed tannins on the final wine tannin content (relative to skin tannins) may also be tentatively attributed to intrinsic differences in the chemical makeup of seed and skin tannins and/or the matrix composition of the must/wine during fermentation and aging. For example, specific components of the wine matrix, such as polysaccharides, can affect the amount of tannin effectively retained in wine [52,53].

**Figure 5:**
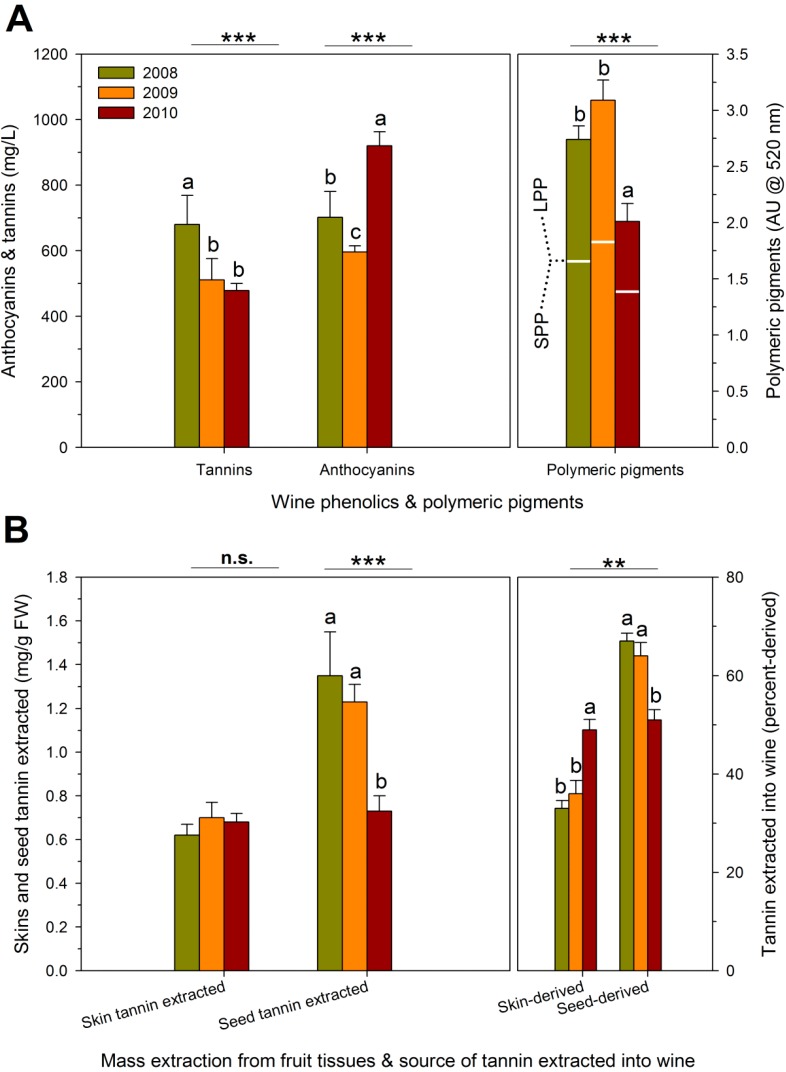
(**A**) Wine phenolics and polymeric pigments; and (**B**) extraction of skin and seed tannins during three growing seasons in field-grown, own-rooted Cabernet Sauvignon grapevines in the Columbia Valley, WA (USA). *, **, and *** indicate significant differences for Duncan test and *p* < 0.05, *p* < 0.01 and *p* < 0.001, respectively. n.s.: not significant.

Figure 5A,B show the anthocyanin, tannin and polymeric content of the finished wines, as well as the amount of skin and seed tannins extracted as affected by the growing seasons. Tannins were highest in 2008 whereas anthocyanins were highest in 2010, with 2009 wines showing intermediate values. Anthocyanin concentration in the fruit was higher in 2010 (Figure 3A), which corresponds with the higher anthocyanin concentration of the 2010 wines. However, although concentration and content of skin and seed tannins were the lowest in the fruit of the 2008 season (Figure 3A,B), the tannin concentration was highest in the 2008 wines (Figure 5A). One possible explanation for this discrepancy can be found in the extent of seed tannin extraction, which was greater (in spite of lower fruit concentration) in 2008 (Figure 5B). The estimated proportion of seed-derived tannins was also greater in 2008 (67%) whereas that of skin-derived tannins was the lowest (33%).

Significant effects of the growing seasons were also observed for the formation and content of polymeric pigments in the wines (Figure 5A). The highest contents occurred in 2008 and 2009 and the lowest in 2010. Both SPP and LPP were also lowest in 2010. This occurred in spite of higher anthocyanin concentrations in the 2010 wines, suggesting that above a certain threshold (clearly reached in the wines of the present experiment), anthocyanins are not the limiting factor for the formation of polymeric pigments. It has been suggested that the formation of polymeric pigments is modulated by the molar proportion of anthocyanins and tannins rather than by the individual concentrations of anthocyanins and tannins [54]. Our data appear to support this idea. However, the dynamic nature of tannin-anthocyanin covalent and non-covalent interactions during maceration and aging may complicate the establishment, based solely on fruit data, of a specific anthocyanin to tannin ratio that would maximize polymeric pigment formation in the wine.

To further explore the potential effect of the wine matrix on wine tannin retention, the amount of unaccounted tannins and the proportion of tannins that were theoretically “bound” to the insoluble wine matrix were calculated (Table 5). These calculations were based on the theoretical extraction of wine tannins (based on what was found in the pomace after maceration), the observed or actual extraction measured in the wines and the initial (total) fruit tannin content. A previous report on Cabernet Sauvignon indicated that the insoluble wine matrix could capture more than 22% of the tannin present in the fruit [55]. In the present experiment, the proportion of bound tannins ranged from 17% to 29%. There was no effect of the RDI regimes on the amount of unaccounted tannins or the proportion of bound tannins. Conversely, there was a clear effect of the growing seasons on these two components: in 2010 there was a significantly lower amount of unaccounted tannins and, accordingly, the lowest proportion of bound tannins. Therefore, the lower tannin content of the 2010 wines was not explained by a higher proportion of bound tannin but rather by a lower extractability of seed tannins (Figure 5B). Similarly, the higher tannin content of the 2008 and 2009 wines was explained by a comparatively higher extractability of seed tannins (Figure 5B), although the proportion of bound tannins was also higher in both years (Table 5). It is also worth noting that both the RDI regimes and the growing seasons affected the proportion of tannin extracted from seeds, whereas none of these two factors affected the proportion of tannins extracted from skins. Additionally, the growing season also altered the proportion of skin- and seed-derived tannins and the proportion of bound tannin.

**Table 5:** Two-way ANOVA of the effect of RDI regime and growing season on the theoretical and observed extraction of wine tannins, the amount of unaccounted tannins and the proportion of bound tannins in finished wines made from fruit of field-grown, own-rooted Cabernet Sauvignon grapevines in the Columbia Valley, WA, USA.

RDI Treatment	Theoretical Extraction (mg/L)	Observed Extraction (mg/L)	Unaccounted Tannins (mg/L)	Proportion Bound Tannins (%) ^#^
IS ^†^	1743 ± 261 b,^‡^	464 ± 15 c	1279 ± 73 a	27 ± 2 a
ED	1747 ± 262 b	538 ± 76 b	1208 ± 192 a	24 ± 4 a
LD	1470 ± 222 c	426 ± 37 c	1044 ± 253 a	22 ± 5 a
FD	2136 ± 454 a	806 ± 140 a	1330 ± 318 a	24 ± 7 a
**Season**				
2008	1972 ± 356 a	680 ± 134 a	1292 ± 236 ab	26 ± 4 a
2009	1935 ± 131 a	517 ± 94 b	1417 ± 72 a	29 ± 1 a
2010	1415 ± 108 a	478 ± 34 b	936 ± 115 b	17 ± 3 b
**RDI × Season interaction**	ns	ns	ns	ns

^†^ IS: industry standard; ED: early deficit (fruit set to *véraison*); LD: late deficit (*véraison* to harvest); FD: full season deficit (fruit set to harvest); ^‡^ Means followed by different letters differ significantly at *p* < 0.05 by Duncan’s new multiple range test; ns = Not significant; ^#^ Calculated relative to the fruit initial tannin content.

### 2.5. Sensory Analysis

**Figure 6:**
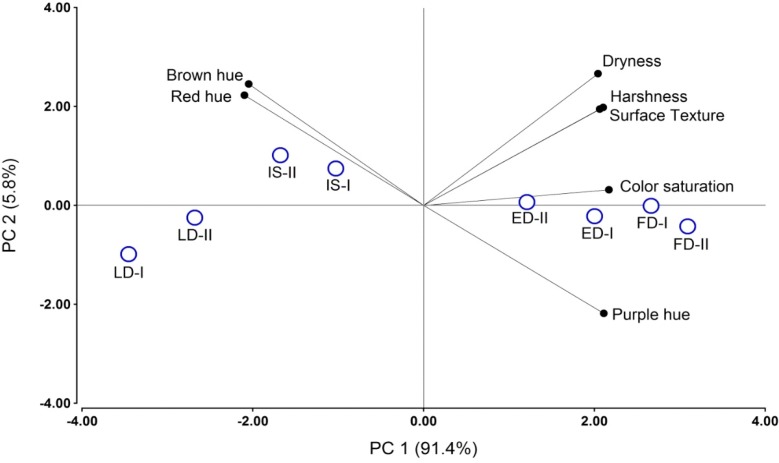
Principal Component Analysis of sensory data of the 2008 wines obtained from field-grown, own-rooted Cabernet Sauvignon grapevines in the Columbia Valley, WA (USA). All the two wine replicates were included in the analysis and are indicated as “I” and “II”. IS: industry standard; ED: early deficit (fruit set to *véraison*); LD: late deficit (*véraison* to harvest); FD: full season deficit (fruit set to harvest).

The 2008 wines were analyzed after 12 months of bottle aging by a trained panel in 2010. The 2008 wines were selected for Descriptive Sensory Analysis (DA) on the basis of being the most representative of an average year from a climatic standpoint. Table 6 shows that there were significant differences among the wines in all seven attributes selected to describe them. The FD wines were the most saturated in color, with higher purple hue, roughness, dryness and harshness, followed by ED wines. On the other hand, IS and LD wines were less saturated in color and with higher brown and red hues. These differences were confirmed by Principal Component Analysis (PCA), in which the FD and ED wines were separated along PC1 from the LD and IS wines (Figure 6). Generally, a strong early (*i.e.*, pre-*véraison*) deficit such as the one applied in the FD and ED regimes resulted in positive sensory effects on wine color saturation [10], probably as a result of a reduction in berry size (*i.e.*, concentration effect for anthocyanins) and its effect on wine anthocyanins. Roughness, dryness and harshness, which also defined the mouthfeel characteristics of FD wines, are tactile attributes related to the wine’s tannin content [56]. The FD wines were the most tannic, especially in 2008 (data not shown). In Merlot wines, the drying mouthfeel was associated with proportionally greater seed tannin extraction [46], which was another feature observed in our FD wines. In a RDI trial in Cabernet Sauvignon, astringency and bitterness defined the wines produced from grapes submitted to a 25% ET_c_ regime. These attributes were in turn correlated with wine tannins, total phenolics and seed-derived tannins [10]. Seed tannins have been considered problematic by winemakers [46], because they are associated with tactile descriptors bearing somewhat negative connotations (e.g., roughness, dryness, harshness). However, it is currently not known if wine tactile sensations such as roughness and dryness are driven by the specific chemical makeup of seed tannins or if they are solely driven by the overall concentration of wine tannins regardless of their composition.

**Table 6:** One-way ANOVA of the effect of RDI regime on sensory attributes assessed by a trained panel (*n* = 9) of Cabernet Sauvignon wines made from fruit of field-grown, own-rooted Cabernet Sauvignon grapevines in the Columbia Valley, WA, USA.

RDI Treatment	Wine Attributes						
	Roughness	Dryness	Harshness	Saturation	Brown Hue	Purple Hue	Red Hue
IS ^†^	9.6 b ^‡^	9.7 b	9.2 b	10.2 b	2.2 b	2.5 a	11.1 b
ED	10.5 bc	10.8 bc	10.3 bc	11.7 c	1.3 a	6.6 b	7.9 a
LD	8.1 a	7.9 a	7.4 a	9.1 a	2.3 b	2.5 a	10.9 b
FD	11.1 c	11.1 c	11.1 c	12.8 d	1.1 a	8.2 b	6.8 a

^†^ IS: industry standard; ED: early deficit (fruit set to *véraison*); LD: late deficit (*véraison* to harvest); FD: full season deficit (fruit set to harvest); ^‡^ Within a column, values followed by the same letter are not significantly different according to Fisher’s LSD test at *p* < 0.05. Evaluations made along a 15 cm unstructured line scale.

During the panel training sessions included in the sensory analysis, it was noted that there were almost no differences in the aroma profile of the wines of the four RDI regimes. This result conflicts with previous reports of the sensory profile of wines produced with different RDI regimes. For example, a previous report indicated that the application of two RDI regimes (70% ET_c_ and 25/100% ET_c_) increased the red and black fruit aroma of Cabernet Sauvignon wines relative to two other RDI regimes (100% ET_c_ and 25% ET_c_) [10]. In Merlot, wines produced with 35% ET_c_ had the most intense spicy flavor and fresh fruit aroma, while 100% ET_c_ produced wines higher in canned vegetal aroma [57]. In Tempranillo, a 25% ET_c_ regime increased floral and fruity aromas and reduced herbaceous aromas in the resulting wines [58]. However, in those studies the wines were submitted to sensory analysis within 3 to 5 months of production, whereas in the present experiment the wines were analyzed after 12 months of bottle aging. It is thus possible that some treatment-specific differences in the aroma profile of the wines originally present shortly after fermentation may have subsided or disappeared with bottle aging. Similar to the results for the phenolic composition of the wines, the FD and ED wines showed similar sensory profiles, and the same was true for the IS and LD wines.

## 3. Experimental Section

### 3.1. Vineyard Site and Experimental Design

The experiment was conducted during the 2008, 2009 and 2010 growing seasons in the Cold Creek vineyard of Ste. Michelle Wine Estates, SE of Mattawa, Washington, WA, USA (latitude 46°57'N, longitude 119°89'W). The vineyard lies in the Columbia Valley AVA and is a source of ultra-premium fruit. Own-rooted *Vitis vinifera* L. cv. Cabernet Sauvignon (clone 8) were planted in 1981 with a vine by row spacing of 2.1 m by 3 m in N-S oriented rows. Vines were trained to a bilateral cordon, spur-pruned to 47 nodes per vine, and shoots were loosely positioned between two foliage wires 30 cm above the cordon. The vineyard was drip-irrigated using pressure-compensated emitters (flow rate 4 L/h) spaced 1.14 m apart. The root zone was irrigated to field capacity before bud break, but irrigation was interrupted before bloom to control shoot growth [3]. Four irrigation regimes were imposed at fruit set (growth stage 27) [59]. The current industry standard (IS) for RDI was used as a control to replenish 70% of full-vine evapotranspiration (70% ET_c_) from fruit set through harvest. The ET_c_ was estimated from a reference crop (grass) evapotranspiration (ET_0_), provided by an on-site weather station (see 3.2. Weather data), and a variable crop coefficient, K_c_ (from ~0.3 at the start of treatments to ~0.8 in early August to ~0.4 by harvest), developed for fully irrigated Cabernet Sauvignon in eastern Washington: ET_c_ = ET_0_ × K_c_[4]. Three more severe RDI regimes were imposed at specific phenological stages. The early-deficit or pre-*véraison* treatment (ED) supplied 30%–35% ET_c_ from fruit set to *véraison* and 70% ET_c_ from *véraison* through harvest. The late-deficit or *post-véraison* treatment (LD) supplied 70% ET_c_ from fruit set to *véraison* and 30%–35% ET_c_ from *véraison* through harvest. The full-season treatment (FD) supplied 30%–35% ET_c_ from fruit set through harvest. The experiment was designed as a randomized complete block with four replicated blocks (*n* = 4) and the irrigation treatments randomly applied (≥6 rows of ≥40 vines each) within each block. The amount of applied water was estimated from drip emitter flow rates and the duration of each irrigation event; the duration was varied by RDI regime and seasonal progression to account for treatment differences and changes in K_c_. Total irrigation water applied during the growing season is reported in Supplemental Figure S2.

### 3.2. Weather Data and Field Measurements

Daily meteorological data were obtained from an on-site weather station located less than 400 m from the experimental site. Growing degree days (GDD) for the period April 1st through October 31st were estimated from daily mean temperatures using a base temperature of 10 °C. All vine-based measurements were taken on four data vines per treatment replicate. Canopy density was assessed each year shortly after *véraison* by the point-quadrat method to estimate leaf-layer number (LLN), as well as the proportion of sun-exposed leaves and clusters [60]. Winter pruning weight (a measure of plant vigor) and cane number and weight were obtained from two vines per treatment replicate. Harvest occurred on 29/9/2008, 21/9/2009 and 14/10/2010. Five vines per treatment replicate were hand-harvested and the clusters counted and weighed. Thirty clusters were taken and put in self-sealing bags, placed on ice and transported to the laboratory where they were counted and weighed.

### 3.3. Winemaking

The grapes were machine-harvested and delivered immediately to the winery. Due to tank volume limitations, adjacent vineyard replicates were combined so that duplicate wines (*n* = 2) were produced from each RDI regime. Due to high Brix in the fruit which can cause stuck or sluggish fermentations, the initial musts were watered back to a target final ethanol content of 14% *v*/*v*. Likewise, titratable acidity was adjusted in the initial musts to a target of 7 g/L using food-grade tartaric acid. Wines were produced and bottled at the Chateau St. Michelle Canoe Ridge facility near Paterson, Washington, WA, USA as previously described (46) with the exception of the lot size which for the present study was ~1000 kg per replicate. The contact time between must and fermentation solids was limited to 7 days in all treatments.

### 3.4. Chemical Analysis Reagents

Bovine serum albumin (BSA, Fraction V powder), sodium dodecyl sulfate (SDS; lauryl sulfate, sodium salt), triethanolamine (TEA), ferric chloride hexahydrate, and (+)-catechin were purchased from Sigma (St. Louis, MO, USA). Malvidin-3-glucoside was purchased from Polyphenols Laboratories (Sandnes, Norway).

### 3.5. Fruit, Wine and Pomace Analyses

For each field replicate, berries were separated from the sample clusters and 250-berry replicates were selected at random. One hundred berries were used to measure total soluble solids, titratable acidity, pH and anthocyanins, and 30 berries were used for tannin analysis. The remaining fruit was divided and stored for other analyses. Measurements of soluble solids (Atago, Tokyo, Japan), titratable acidity (TA) and pH (DL50 Autotitrator, Mettler-Toledo, Columbus, OH, USA) were taken at the time of harvest. Anthocyanins were measured as described [61,62]. Fruit, pomace and wine tannins were also analyzed as previously described [62] using protein precipitation, and were standardized using catechin equivalents (CE). Total iron reactive phenolics in the wine were analyzed according to [63]. Large polymeric pigments (LPP) and small polymeric pigments (SPP) were measured in the wine according to [61].

For tannin analysis in the fruit, four replicates of 30 berries each were dissected into skins and seeds and extracted separately with 70% acetone as previously described [62]. A Büchi Syncore Polyvap (Flawil, Switzerland) equipped with a 24-well heat block for 50-mL tubes was used to remove the acetone under reduced pressure at 38 °C. Skins and seeds from pomace were separated and used to reconstruct eight 30-berry replicates to analyze tannins recovered in the skins and seeds after maceration. Extraction and solvent removal of the pomace skins and seeds was performed as described elsewhere [46,51].

### 3.6. Sensory Analysis

The 2008 wines were analyzed by descriptive analysis after ~1 year of bottle aging as previously described [64]. A prescreening of the wines by four experienced wine tasters ensured that the wines were different enough to justify a descriptive analysis and also that they were free of sulfur-like or other off-odors.

#### 3.6.1. Materials

Six-*n*-propylthiouracil (PROP) was obtained from Sigma Aldrich (St. Louis, MO, USA). Quinine sulfate, HCl and H_2_O_2_ were purchased from J.T. Baker (Phillipsburg, NJ, USA). Ammonium aluminum sulfate (alum) was obtained from McCormick & Co. (Hunt Valley, MD, USA). Pomegranate juice (POM Wonderful, Los Angeles, CA, USA), NaCl (Safeway Select, Boise, ID, USA) and unsalted crackers (Nabisco, East Hanover, NJ, USA) were obtained from a local grocery store. Commercial wines were used to demonstrate to panelists the different astringency sub-qualities and color components and were obtained through a local wine store. Clear ISO wineglasses (ISO 3591:1977) were used during the training and formal evaluation sessions.

#### 3.6.2. Selection and Training of Sensory Panelists

A pool of potential panelists was recruited from the Washington State University campus (Pullman, WA, USA). Candidates were screened for bitterness sensitivity to PROP following a published protocol [65] and for visual disorders using Ishihara images [66]. Bitter-insensitive panelists, also known as non-tasters, were eliminated from the pool of candidates. None of the selected panelists had color vision deficiencies, and the final panel was composed of 10 individuals (four males and six females) aged between 21 to 30 years. Panelists received minimal information about the nature of the study and signed an informed consent form approved by the WSU Review Board for human subject participation.

During the training and evaluation sessions a 15-cm line scale was used, labeled with terms ‘low’ and ‘high’ at the 1-cm and 14-cm mark from the left-end of the scale, respectively. Eight training sessions, each lasting between 45 min to 1 h were conducted over a period of six weeks. For terminology alignment, three astringency sub-attributes were defined by consensus and demonstrated with commercial wines. *Roughness/surface texture* was defined as the force of friction experienced by rubbing the tongue against the hard palate with the wine in mouth after swishing the wine around the entire mouth cavity for 5 s. *Dryness* was defined as the drying sensation around the upper and lower gums 5 s after expectorating the wine [56]. *Harshness* was defined as the combined effect of astringency and bitterness [56]. The *dryness* and *harshness* standards were prepared at ‘low’, ‘medium’ and ‘high’ levels, representing anchors located at 1 cm, 7.5 cm and 14 cm from the left-end in the 15-cm unstructured scale. For the *dryness* standard, water solutions of alum were made at a rate of 250 mg/L (low), 875 mg/L (medium) and 1500 mg/L (high) 5 h prior to the sessions. For the bitterness component of the *harshness* attribute, water solutions of quinine sulfate were prepared at a rate of 5 mg/L (low), 20 mg/L (medium) and 35 mg/L (high) 5 h prior to the sessions. For the saturation color standard, successive dilutions of a Syrah wine from the 2009 harvest produced at the WSU Prosser winery facility were made with deionized filtered water (18.2 MΩ·cm resistivity) 5 h prior to the training sessions. For the brown hue standard, 5 mL of H_2_O_2_ was added to 250 mL of the Syrah wine 5 h prior to the training sessions. The red hue standard was demonstrated with pomegranate juice, and for the purple hue standard 250 mL of the Syrah was acidified with 1.0 mL 12.1 N HCl.

#### 3.6.3. Formal Evaluation Sessions

Wines and their replicates were presented monadically and evaluated twice during four consecutive formal evaluation sessions, following a randomized balanced block design. Panelists assessed the wines in individual booths (20 ± 2 °C) in the Sensory Facility of the Department of Food Science and Human Nutrition, WSU Pullman. Color components were evaluated under natural daylight. To avoid perceptual bias due to color, mouthfeel attributes were evaluated under red light. Thirty mL aliquots of wine at room temperature were poured into wineglasses coded with three-digit random numbers and covered with aluminum lids to trap volatiles. For the assessment of mouthfeel attributes, panelists were instructed to chew one cracker, rinse with deionized water, and wait at least 4 min between samples during which they assessed color components of another sample. Results were collected in ballots with responses decoded manually in centimeters and entered into an Excel spreadsheet (Microsoft, Redmond, WA, USA).

### 3.7. Data Analysis

Vine-related data were analyzed by two-way analysis of variance (ANOVA) to test for interactions between treatments and years. Duncan’s multiple range test was used to compare differences among significant means within years or treatments with a 5% level for rejection of the null hypothesis. Field, grape and wine data were analyzed using Statistica (version 10, StatSoft, Tulsa, OK, USA). For the sensory analysis, panelists’ performance was checked with the PanelCheck software [67], and on the basis of these analyses, one outlier was eliminated (final *n* = 9). The trained panel data were first analyzed by two-way mixed-effects ANOVA with replication, considering panelist as random effect and wine treatments as fixed effects, using XLSTAT ver. 2011. Results are summarized in Supplemental Table 1. Separation of the means was accomplished by one-way ANOVA using Fisher’s LSD test with significance established as *p* < 0.05. Principal components analysis was conducted using the correlation matrix without rotation, using the Infostat statistical package (version 2012, Córdoba, Argentina).

## 4. Conclusions

The present work examined the effect of four RDI regimes on the basic and phenolic chemistry of Cabernet Sauvignon grapes and wines over three consecutive growing seasons in eastern Washington (USA). The sensory profile of the 2008 wines was also determined. The growing seasons in this study experienced very different weather conditions, providing a unique opportunity to evaluate potential interactions between RDI regimes and seasonal effects. From an agronomic perspective, reductions in water supply were as high as 67% in FD relative to IS, but crop yields were reduced by up to 37%. This reduction in yields was due to the effect of pre-*véraison* RDI on berry weight. Berry phenolics were affected both on a concentration basis and on a per berry basis. The RDI regimes mostly affected the concentration of skin and seed phenolics, suggesting that the impact of these techniques is rather indirect and based on a reduction of berry size. Indeed, there was no apparent effect of any of the RDI regimes on seed and skin tannin content, suggesting that tannin biosynthesis is not altered by RDI. The exception seems to be the content of anthocyanins under conditions of continuous water stress such as the one imposed in FD, in which the amount of anthocyanins increased. On the other hand, the content of phenolics was season-dependent, implying that different growing seasons are associated with specific biosynthetic effects that alter the phenolic content and, potentially, extraction and retention into wine.

The basic chemistry of the wines was generally not affected by the RDI regimes, and overall, wine phenolics mirrored differences in fruit phenolics, particularly for anthocyanins. The FD wines had higher concentrations of anthocyanins, tannins and large polymeric pigments. Both the RDI regimes and the growing seasons affected the proportion of tannin extracted from seeds, whereas none of these two factors affected the proportion of tannins extracted from skins. The growing season also affected the proportions of skin- and seed-derived tannins and the proportion of bound tannins.

The sensory analysis showed that the FD wines were defined by mouthfeel descriptors such as roughness, dryness and harshness, related to the wine tannin concentration, and wine color saturation, which was related to the wine anthocyanin concentration.

Over the three years of this study, the FD and ED regimes seemed to yield fruit and wines more enriched in phenolic composition than those of the IS and LD regimes, with the additional advantage of reducing water usage. However, these apparent benefits need to be balanced out with reductions in crop yields and potential long-term effects associated with pre-*véraison* water deficits.

## Supplementary Materials

Supplementary materials can be accessed at: http://www.mdpi.com/1420-3049/20/05/7820/s1.
