# Supplementary material for: Regulated Deficit Irrigation Alters Anthocyanins, Tannins and Sensory Properties of Cabernet Sauvignon Grapes and Wines
Source: Molecules. 2015 Apr 29;20(5):7820–44. doi: 10.3390/molecules20057820 (PMC6272144; doi:10.3390/molecules20057820)
Supplement: Supplementary file 1 [file molecules-20-07820-s001.pdf]

Supplementary Materials

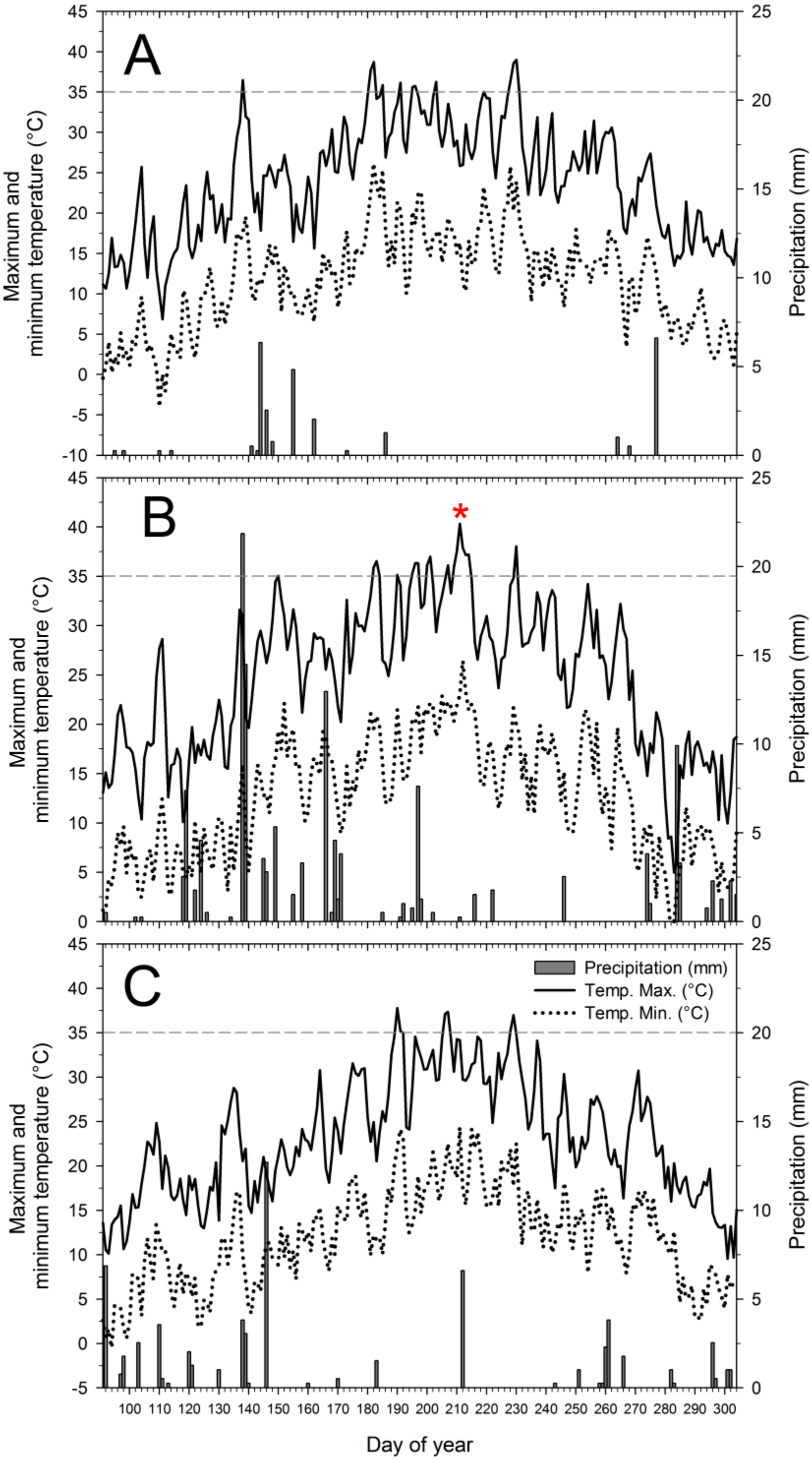

Figure S1. Maximum and minimum temperatures and precipitation during three growing season from April 1st (Day 91) to October 31st (Day 304) registered in the vineyard during three growing seasons in field-grown, own-rooted Cabernet Sauvignon grapevines in the Columbia Valley, WA (USA). (**A**) 2008 season; (**B**) 2009 season; (**C**) 2010 season. The grey dotted line indicates 35 °C. The red asterisk indicates one episode of 40.3 °C in 2009.

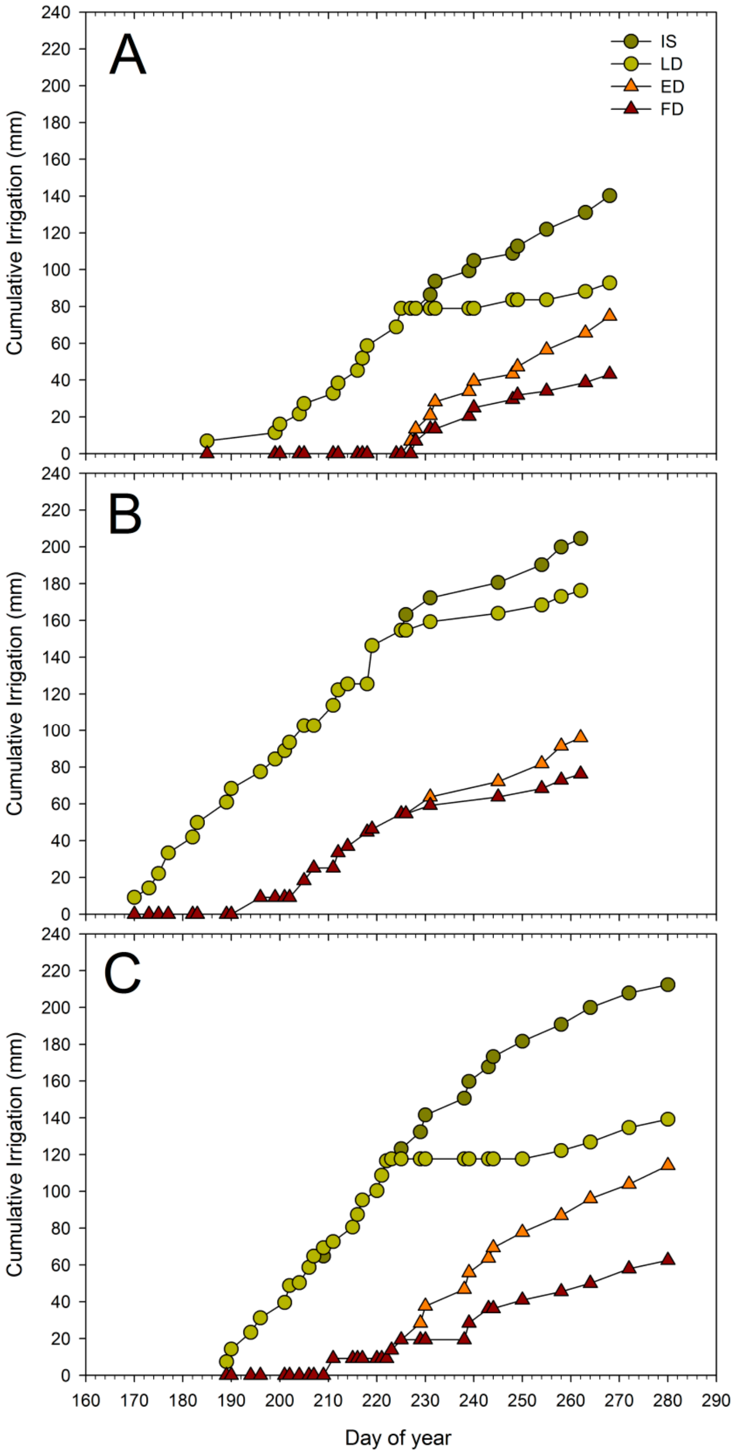

Figure S2. Cumulative irrigation applied during three growing seasons in field-grown, own-rooted Cabernet Sauvignon grapevines in the Columbia Valley, WA (USA). (**A**) 2008 season; (**B**) 2009 season; (**C**) 2010 season. IS: industry standard; ED: early deficit (fruit set to véraison); LD: late deficit (véraison to harvest); FD: full season deficit (fruit set to harvest).

Table S1. Degrees of freedom and F-ratios from a two-way ANOVA of trained panel evaluation of four wines.

Source of variation	Degree of freedoms	Wine Atributes						
		Roughness	Dryness	Harshness	Saturation	Brown Hue	Purple Hue	Red Hue
Panelist	8	**2.37** [a]	0.64	0.66	**3.84**	**11.32**	2.01	**11.29**
Wine	3	**10.19**	**11.03**	**14.19**	**80.48**	**7.64**	**19.42**	**17.89**
Panelist × Wine	24	1.02	0.74	0.80	1.23	1.24	0.43	1.06

[a] Significant values (Fisher's LSD test, $p < 0.05$) are shown in bold underlined letters.
